# Spatial habitats from multiparametric MR imaging are associated with signaling pathway activities and survival in glioblastoma

**DOI:** 10.18632/oncotarget.22947

**Published:** 2017-12-05

**Authors:** Katherine Dextraze, Abhijoy Saha, Donnie Kim, Shivali Narang, Michael Lehrer, Anita Rao, Saphal Narang, Dinesh Rao, Salmaan Ahmed, Venkatesh Madhugiri, Clifton David Fuller, Michelle M. Kim, Sunil Krishnan, Ganesh Rao, Arvind Rao

**Affiliations:** ^1^ Department of Medical Physics, The University of Texas Graduate School of Biomedical Sciences, Houston, TX, USA; ^2^ Department of Statistics, The Ohio State University, Columbus, OH, USA; ^3^ Bioinformatics and Computational Biology, The University of Texas MD Anderson Cancer Center, Houston, TX, USA; ^4^ Cancer Biology, The University of Texas MD Anderson Cancer Center, Houston, TX, USA; ^5^ Texas Academy of Math and Science, Denton, TX, USA; ^6^ School of Engineering and Applied Sciences, Columbia University, New York City, NY, USA; ^7^ Debakey High School for Health Professions, Houston, TX, USA; ^8^ Radiology, University of Florida, College of Medicine, Jacksonville, FL, USA; ^9^ Diagnostic Radiology, The University of Texas MD Anderson Cancer Center, Houston, TX, USA; ^10^ Neurosurgery, Tata Memorial Hospital, Mumbai, India; ^11^ Radiation Oncology, The University of Texas MD Anderson Cancer Center, Houston, TX, USA; ^12^ Neurosurgery, The University of Texas MD Anderson Cancer Center, Houston, TX, USA; ^13^ Radiation Oncology, The University of Michigan, Ann Arbor, MI, USA

**Keywords:** imaging-genomics analysis, image-derived spatial habitat, glioblastoma, signaling pathway activity, Dirichlet regression

## Abstract

Glioblastoma (GBM) show significant inter- and intra-tumoral heterogeneity, impacting response to treatment and overall survival time of 12-15 months. To study glioblastoma phenotypic heterogeneity, multi-parametric magnetic resonance images (MRI) of 85 glioblastoma patients from The Cancer Genome Atlas were analyzed to characterize tumor-derived spatial habitats for their relationship with outcome (overall survival) and to identify their molecular correlates (i.e., determine associated tumor signaling pathways correlated with imaging-derived habitat measurements). Tumor sub-regions based on four sequences (fluid attenuated inversion recovery, T1-weighted, post-contrast T1-weighted, and T2-weighted) were defined by automated segmentation. From relative intensity of pixels in the 3-dimensional tumor region, “imaging habitats” were identified and analyzed for their association to clinical and genetic data using survival modeling and Dirichlet regression, respectively. Sixteen distinct tumor sub-regions (“spatial imaging habitats”) were derived, and those associated with overall survival (denoted “relevant” habitats) in glioblastoma patients were identified. Dirichlet regression implicated each relevant habitat with unique pathway alterations. Relevant habitats also had some pathways and cellular processes in common, including phosphorylation of STAT-1 and natural killer cell activity, consistent with cancer hallmarks. This work revealed clinical relevance of MRI-derived spatial habitats and their relationship with oncogenic molecular mechanisms in patients with GBM. Characterizing the associations between imaging-derived phenotypic measurements with the genomic and molecular characteristics of tumors can enable insights into tumor biology, further enabling the practice of personalized cancer treatment. The analytical framework and workflow demonstrated in this study are inherently scalable to multiple MR sequences.

## INTRODUCTION

Glioblastoma (GBM), the most commonly diagnosed malignant brain tumor in adults [1], has a poor prognosis, with a median survival of only 12-15 months and a high rate of recurrence [2]. Poor prognosis and overall survival (OS) is attributable to the marked inter- and intra-tumoral genetic heterogeneity of GBM tumors [3–6]. Magnetic resonance imaging (MRI) holds great potential for characterizing the phenotypic heterogeneity of GBMs by inferring this from textural information and intensity variations in radiological images. Common techniques such as gadolinium contrast-enhanced T1-weighted imaging highlight perfusion variations in tumor images and advanced image-texture analysis may be able to characterize signal intensity variations within tumors. Texture analysis has many applications in medical image processing and provides one approach to quantify the distribution of gray-level patterns such as homogeneity, entropy, etc., within a set of imaging data [7]. Multiple methods for assessing imaging features and characterizing pixel intensity distributions by quantifying gray levels have been described [8–11]. These methods allow for rigorous and reproducible derivation of detailed, pertinent information, and have been used to analyze MRI features, such as apparent diffusion coefficient, 2-dimensional (2D) spatial habitats [12], and texture features. These characteristics correlate with the grade of disease, patient survival, response to chemotherapy, and genetic and epigenetic status [12–18]. With recent advances in radiomics and radiogenomics (or imaging-genomcis), molecular and genetic heterogeneity can be inferred from MRI features by correlating imaging datasets with corresponding molecular and clinical information.

Several studies have correlated specific features seen in MRI of GBM with patient survival and molecular subtype. However, these investigations have been restricted to 1 or 2 particular MRI sequences and typically considered only features from a single 2D slice [12, 16, 17]. In contrast to these single-sequence, slice-by-slice analyses, radiologists review all acquired MRI sequences in their assessments. Additional research is needed to develop methods for extracting computational radiological features from full multiparametric MR imaging sets. To this end, this study focuses on using four MR sequences to understand the heterogeneity of a tumor region.

We hypothesized that by performing a 3D volumetric analysis of commonly available MRI sequences, we could identify particular imaging habitats [17] correlated with both patient overall survival status and identify key genetic pathways associated with such habitats in GBM. Comprehensive analysis of imaging data could improve the predictive power of this approach and provide novel insights to aid clinical decision-making.

## RESULTS

### Identifying imaging habitats

**Figure 1:**
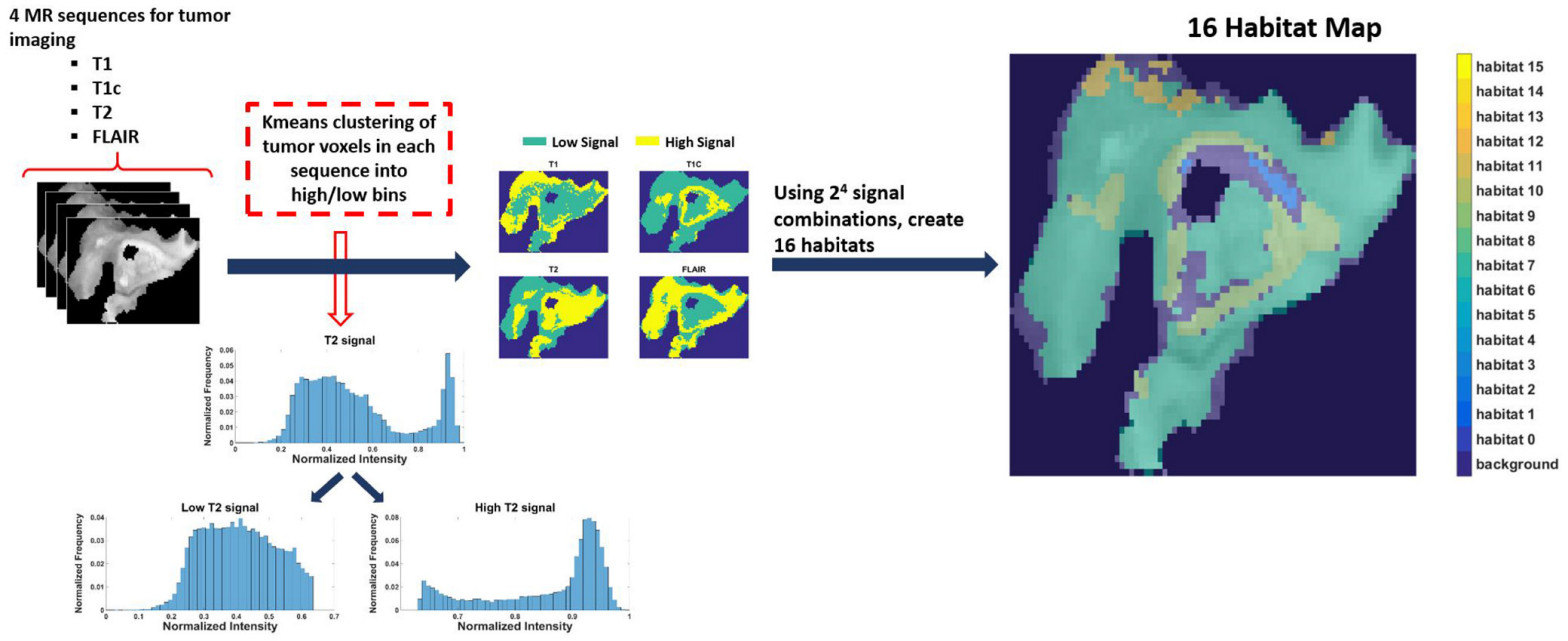
The process of generating 16 spatial habitats Based on 4 MR sequences (multi-parametric MRI scans), classify each voxel within the tumor volume into high and low categories via kmeans clustering. With 16 (2^4^) signal combinations across the 4 sequences (i.e. 0000-1111), every voxel in the tumor volume can be identified uniquely. The resultant habitat map shows the spatial heterogeneity within tumor.

Eighty-five patients with GBM identified in the Cancer Genome Atlas who had imaging, clinical, and genomic data available were included in this study. Table 1 shows the patient and respective tumor characteristics within 85 TCGA-GBM cases. Four pre-operative MR scans were obtained for each case: Pre-contrast T1-weighted (T1) image, post-gadolinium T1 (T1c) image, T2-weighted (T2) image, and T2 fluid-attenuated inversion recovery (FLAIR) image. Following skull stripping, rigid registration, and automated segmentation using Brain Tumor Image Analysis (BraTumIA) software [19], we grouped tumor voxels into high and low signal bins for each of the 4 MRI sequences (FLAIR, T1, T1c, and T2) via Kmeans clustering. Across these 4 MR sequences, this leads to the identification of 16 imaging habitats [17] (i.e 2^4^ combinations) based on unique combinations of these high and low signals. The habitats were assigned labels from 0 to 15, with “0” being low-intensity in all 4 acquisition modalities (i.e., FLAIR=0, T1=0, T1c = 0, T2 = 0) and “15” being high-intensity in all 4 (i.e., FLAIR=1, T1=1, T1c = 1, T2 = 1). For instance, habitat 2 represents low intensity in FLAIR, T1, and T2 with high intensity in T1c. Figure 1 describes the process for obtaining these 16 imaging habitats.

**Table 1:** Clinical data from 85 TCGA patients with primary GBM

Patient or tumor characteristics	Number of patients (%)
Age	
Median	59 years
Range	18-84 years
Sex	
Male	65 (%)
Female	35 (%)
Overall survival median (range)	11.7 (0.5 – 90.9) months
Disease-free survival median (range)	6.64 (0.7 – 57.7) months
IDH1 mutation	
Yes	2 (2)
No	83 (98)

### Identifying important and significant imaging habitats using clinical data

**Figure 2:**
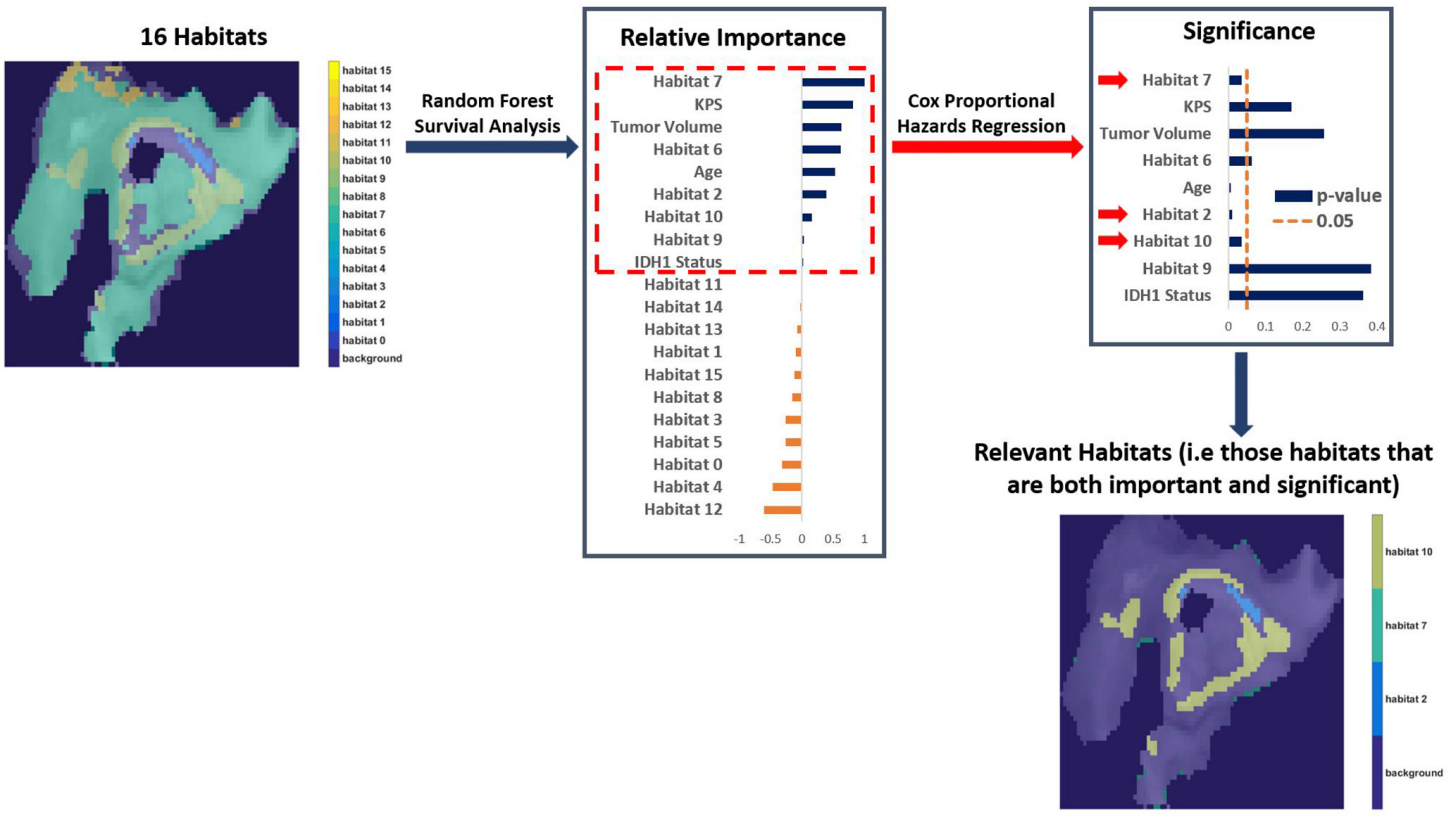
The process of finding important and significant (designated “Relevant”) habitats After adjusting for clinical covariates (age, Karnofsky performance score, tumor volume, and IDH1 mutation status), we identified important habitats (positive variable importance) via Random Forest survival analysis. Those habitats were then assessed for significance via Cox Proportional Hazards Regression to determine overall survival (OS). Only habitat 2,7, and 10 are both important and significant (i.e “relevant”) in determining OS.

Random survival forest modeling and Cox proportional hazards regression analysis determined imaging habitats 2,7, and 10 to be both important (+ve variable importance) and significant (p-value < 0.05) for determining OS after adjustment for covariates of age, Karnofsky performance score, tumor volume, and IDH1 mutation status (*P* < 0.05, Supplementary Table 1). These three habitats were thus designated as “relevant” habitats. The process of acquiring these relevant habitats is described in Figure 2. They represent distinct tumor sub-regions of clinical relevance to outcome in GBM, after adjusting for clinical covariates. Their relevance, coupled with the availability of matched genomic data for these patients, enables us to study the molecular mechanisms (pathway activities) associated with the presence of these habitats in GBM.

### Associating relevant imaging habitats with canonical tumor sub-volumes and genetic pathways

We used the pair-wise Spearman rank test to correlate the amount of each relevant habitat in the tumor with the amount of necrotic, enhancing, non-enhancing, and edema regions within the tumor (Table 2). In particular, habitat 2 was also associated with necrosis (p = 0.0172). For habitat 7 and 10, there was no specific association with canonical tumor sub-volumes, suggesting the need for deeper examination of their physiology and molecular context.

**Table 2:** Pair-wise Spearman correlation p-values for proportions* of relevant habitats (i.e. those that are important & significant) associated with canonical tumor sub-volumes (edema, necrosis, enhancing, and non-enhancing regions)

Habitat	Necrosis	Non-enhancing	Enhancing	Edema
2	0.0172	0.1003	0.2274	0.9239
7	0.1112	0.3356	0.2670	0.1680
10	0.0937	0.8381	0.2670	0.1680

^*^ Habitat proportions are the fraction of tumor pixels belonging to each imaging habitat computed for each patient.

To study the relationships between relevant habitat proportions and molecular pathway data [20], we performed Dirichlet regression analysis. Table 3 lists the key genetic pathways that are most strongly correlated with the relevant habitats. In particular, habitat 2 was positively associated with positive regulation of NFκB transcription-factor activity; while negatively associated with dendrite morphogenesis. Habitat 7 is correlated positively with DNA damage response signal transduction resulting in induction of apoptosis and macrophage activation. Further, habitat 7 was correlated negatively with immune cell activity (monocyte differentiation). Habitat 10 shows positive association with activity of signal transducers and activators of transcription-1 (STAT-1) and Natural killer cell activation, while showing negative correlated with ion channel activity (potassium channel inhibitor activity and voltage gated calcium channel activity).

**Table 3:** Genetic pathways that are associated (top five in magnitude) with important-significant (i.e “relevant”) imaging habitats

	Habitat 2	Habitat 7	Habitat 10
**Upregulated Pathways**	Positive regulation of NFκB transcription-factor activity	DNA damage response signal transduction by p53 class mediator resulting in induction of apoptosis	Positive regulation of tyrosine phosphorylation of STAT-1
	Sphinganine 1-phosphate	Macrophage activation	Natural killer cell activation
	T cell proliferation during immune response	Neuron projection morphogenesis	Wnt receptor signaling pathway
	Cytokine production during immune response	Positive regulation of tyrosine phosphorylation of STAT-1	Mol beta2 estradiol
	T helper 2 cell differentiation	Natural killer cell activation	cAMP biosynthetic process
**Downregulated Pathways**	Dendrite morphogenesis	Monocyte differentiation	Potassium channel inhibitor activity
	Calcineurin A alpha beta B1	Response to DNA damage stimulus	Voltage gated calcium channel activity
	Proteasomal ubiquitin dependent protein catabolic process	T-cell differentiation	Positive regulation of cyclin dependent protein kinase activity
	IGF 1R heterotetramer	Cell morphogenesis	Regulation of S phase of mitotic cell cycle
	Negative regulation of DNA binding	Actin cytoskeleton reorganization	Schwann cell development

## DISCUSSION

Through analysis of multi-parametric MR imaging, we have identified tumor sub-regions with unique combinations of gray-level intensities from each of the four MR modalities included in this study. From these 16 habitats, relevant (i.e. important and significant) habitats were identified based on association with OS status, and further, correlated with morphological and pathological characteristics of tumor, such as leading edge, infiltrating tumor into normal brain, edema, and enhancement around lesion edge. For example, habitat 10 was associated with canonical tumor sub-volumes like edema, peripheral tumor tissue, and enhancement around lesion edge.

Our analysis reveals that the intratumoral abundance of these habitats are associated with outcome. Delineating the extent and location of these aggressive habitats can have implications for delivery of radiotherapy (boosting RT to aggressive habitat areas), surgical resection (an aggressive habitat not impinging on an eloquent area is amenable for possibly complete resection). Additionally, tracking/monitoring the growth of aggressive habitat subvolumes can provide a deeper understanding of disease evolution/recurrence instead of just gross tumor volume. The utility of defining heterogeneity in glioblastoma thus is closely related with prognosis and overall survival of patients [1–4]. Hence, defining such phenotypic heterogeneity is directly relevant for treatment planning, surgical intervention, disease monitoring, and prognosis estimation in the clinic.

Dirichlet regression implicated each relevant habitat with pathway alterations due to unique upstream transcriptional regulators and signaling activity. Imaging-derived habitats also showed some common cellular processes and pathway activity, such as natural killer cell and STAT-1 signaling. These findings are consistent with hallmarks of cancer [21, 22], such as avoiding cell death (natural killer cell) and inducing angiogenesis (tyrosine phosphorylation of STAT-1).

Recent literature supports the relationships inferred between clinical pathologies for habitats (Table 4) and pathway alterations (Table 3). For instance, it is reported that that overexpression of inflammatory cytokines is linked with the leading edge of the gliomas. This is consistent with our findings (positive association of cytokine production) in habitat 2, which is associated with the leading edge of the tumor [23]. Furthermore, it is well known that glioblastoma is infiltrated by diverse immune cells including macrophages [24], which might explain the positive association of macrophage activation and habitat 7. It is also reported that potassium channel inhibitor activity plays a critical role in cell proliferation and cell swelling in neuroblastoma and gliomas [25]. Further, Sforna et al. [26] reported that calcium ions channels play a role in swelling in GBM. These reports are consistent with our clinical pathology interpretation, edema, for habitat 10.

**Table 4:** Interpretation of important-significant (i.e. relevant) imaging habitats based on multiparametric MR: ‘0’ denotes low in signal intensity and ‘1’ denotes high in signal intensity

Habitat number	Enhancement combination				Clinical pathology
	FLAIR	T1	T1C	T2	
2	0	0	1	0	Leading edge of the tumor
7	0	1	1	1	Overall tumor mass including leading edge and infiltrating tumor into normal brain
10	1	0	1	0	Edema, peripheral tumor tissue, enhancement around lesion edge

Our study has some potential limitations. This retrospective study relied on publicly available imaging data acquired across multiple clinical sites. Thus there were some variations in imaging acquisition/sequence parameters (e.g., Relaxation Time and Echo Time) and hardware (e.g., magnetic field strength and receiver coil geometry). In order to mitigate these effects, and in accordance with standard practice, intensity normalization and registration were applied to each image prior to habitat analysis. However, any remaining intensity variations could have potentially affected initial tumor segmentation. A systematic study with standardized image acquisition protocols is needed in order to validate the robustness of these imaging-derived habitat characteristics. Another aspect of uncertainty in this study is derived from the patient outcome data. Because these patients were treated at different institutions, aspects of their treatment regimens may not have been fully standardized, which could potentially affect patient OS duration. Additionally, an inherent challenge with all genomic information derived from tumor specimens is the inability to account for spatial heterogeneity of genomic alterations and tumor cell clones. As with the quality of the imaging data described above, the robustness of the genomic data is also subject to similar uncertainties that can influence the overall generalizability of our conclusions. Also, the presented relationships between habitats and genetic pathway alterations are inferred based on statistical regression methods. These findings could potentially form the basis for targeted perturbation experiments in-vivo to illuminate the mechanistic or causative nature of the relationships between habitats and pathway activity. While outside the scope of this study, research efforts in this direction [27, 28] are essential before these associations can be exploited therapeutically. However, the process of habitat inference and its association mining for prognostic intent can be done quite readily in the current clinical scenario since these 4 MR sequences (T1, T1c, T2, FLAIR) are used routinely.

## MATERIALS AND METHODS

### Datasets

One hundred patients with primary GBM were identified from The Cancer Genome Atlas (TCGA). Patient image data and corresponding clinical data were extracted from The Cancer Imaging Archive [35]. All patients had undergone routine MRI prior to surgery and treatment. Pre-contrast T1-weighted (T1) images, post-gadolinium T1 (T1c) images, T2-weighted (T2) images, and T2 fluid-attenuated inversion recovery (FLAIR) images were acquired. Patient genomic and molecular data were obtained from cBioPortal [36] and included data on genetic pathway activation (PARADIGM scores [20]). This study abided by the TCGA data use agreement and was Institutional Review Board-exempt.

Of the initial 100 GBM patients identified, 85 patients had a complete set of imaging, clinical, and genomic data available. These patients were separated into 2 survival groups: OS (OS) ≥ 12 months and OS < 12 months. The acquisition parameters for the four MRI sequences used in this study are as follows – T1 (echo time: 15-8.5 ms, relaxation time: 642-400 ms), T1 post contrast (echo time: 15-8.5 ms, relaxation time: 700-400 ms), T2 (echo time: 120-30 ms, relaxation time: 6000-2140 ms) and FLAIR (echo time: 150-90 ms, relaxation time: 11000-6000 ms).

### Image processing, image registration and segmentation

For our study, Brain Tumor Image Analysis (BraTumIA) software [19] was used to perform image preprocessing steps (skull stripping and rigid registration), followed by automated segmentation of normal structures (cerebrospinal fluid, gray matter, and white matter) and diseased tissue (necrosis, edema, non-enhancing tissue, and enhancing tissue) [37].

### Image habitat analysis

Patient images and the BraTumIA-derived segmentation masks were loaded into MATLAB for habitat analysis. For habitat analysis, we used only the regions of the MR image associated with tumor (i.e. necrosis, edema, non-enhancing tissue, and enhancing tissue). These BraTumIA segmentation labels were combined to create one binary mask of the tumor region for each patient. The masks were applied to each scan to extract intensity values from the tumor. The tumor intensity values were first scaled relative to gray matter and white matter intensity and then subsequently, linearized (scaled from 0 to 1) based on the maximum and minimum in each image. Following Zhou et.al [17] K-means clustering was applied to each MR sequence type (FLAIR, T1, T1c, and T2) across all patients to derive sequence-specific, intensity thresholds that separate high-intensity pixel values from low-intensity pixel values. Each sequence image for each patient was then dichotomized into sub-regions of high or low enhancement (1 or 0, respectively). The combinations of high and low enhancement for each patient resulted in 16 possible imaging habitats, where habitat 0 is represents regions with low-intensity (FLAIR = 0, T1 = 0, T1+C = 0, T2 = 0), habitat 3 represents regions with characteristics (FLAIR = 0, T1 = 0, T1+C = 1, T2 = 1), and habitat 15 represents regions having high-intensity in all acquisitions (FLAIR = 1, T1 = 1, T1+C = 1, T2 = 1). A 3D spatial representation of the imaging habitats was created using these labels. Subsequently, habitat proportions, the fraction of tumor pixels belonging to each imaging habitat, were calculated for each patient. The process of obtaining 16 habitats is described in Figure 1.

### Associating imaging habitats with clinical and genomic data

Statistical analysis was performed using R statistical software (R Foundation, Vienna, Austria) with the “survival” (v 2.38-2), “randomForestSRC” (v 2.0.5), and “DirichletReg” (v 0.6-3) packages. Random survival forest (RSF) regression was applied to the imaging habitat proportions to determine if any habitats were associated with OS [16, 38, 39]. The random forest regression identified a subset of habitats that were deemed “important.” Cox proportional hazard regression analysis was then used to determine the association of these (RSF-derived) important habitats with OS (p-values less than 0.05) [40–42], after adjusting for clinical covariates (age, volume, karnofsky score: KPS, IDH1 mutation). Image habitats that were deemed important based on random survival forest regression, and significant from the Cox proportional hazard regression were designated “relevant”. The process of obtaining relevant habitats is depicted in Figure 2. These relevant habitats were then correlated with BraTumIA-derived tumor volume segmentations (edema, necrosis, enhancing regions, and non-enhancing regions) via pair-wise Spearman rank correlation tests. In addition, Dirichlet regression was applied to each relevant habitat to determine if that habitat was associated with pathway activation (PARADIGM) scores. This analysis generated molecular signatures associated with each relevant imaging habitat. The significance of the Dirichlet regression for each pathway was determined by calculating a Bonferroni-adjusted *P* value, and the strength of association was determined from the absolute value of the Dirichlet regression coefficient.

## CONCLUSIONS

This study has elucidated a nuanced relationship between imaging habitats derived from MR images, clinical characteristics, and molecular data in a cohort of GBM patients. The present study implemented an automated methodology that parallels the traditional practice of a neuroradiologist by considering the totality of 3D imaging data (i.e. across multiple MR sequences) from each patient. This technique represents an advance in imaging-genomics. Whereas previous studies derived associated imaging features and genomic data from 2D imaging datasets [12, 29, 30], this study adopted a 3D approach, which is consistent with the progress of the field towards studying 3D image features [10, 31–34] for imaging-genomic analysis. Our approach utilized a multi-parametric representation of 3D image features derived from different imaging sequences overlaid upon each other to generate an imaging phenotype that predicts OS duration, after adjusting for clinical covariates.

This work revealed associations between MRI-derived habitats and oncogenic molecular mechanisms in patients with GBM. The analytical framework and workflow demonstrated in this study are inherently scalable to any number of MR sequences. The four MRI sequences used, FLAIR, T1, T1+C, and T2, are readily available on most modern scanners and used routinely in clinical practice. More advanced sequences such as susceptibility-weighted imaging, diffusion-weighted imaging, or MRI spectroscopy could be incorporated into this framework in a fairly linear manner (with ‘k’ MR sequences, you have 2^k^ habitats). Furthermore, with appropriate imaging and tumor segmentation, this workflow could accommodate data from other tumor types and/or other anatomical sites.

Pathway analysis of the molecular data associated with each habitat showed significantly altered molecular pathways, supporting the hypothesis that each radiographically-distinct tumor habitat is associated with distinct molecular characteristics. Some of these altered pathways pertain to angiogenesis, while others correlate with signaling pathways known to be co-opted by tumor cells to facilitate their avoidance of cell death. The relationships elucidated by this study between imaging habitats and underlying biology may offer additional information regarding the patient's disease state, complementing inference based on genomics alone. Future work will extend this analysis by considering tumor location, extent of resection, methylguanine-DNA methyltransferase (MGMT) promoter methylation status, as well as extending this work to other tumor types and anatomical sites. A systematic assessment of the impact of acquisition parameter variability on robustness of imaging habitats will also need to be performed before clinical adoption. These results also lay the groundwork for investigations of targeted hypotheses from MRI-guided biopsies [27, 28] in GBM with corresponding genomic analyses to confirm and validate these phenotypic-genomic relationships.

## SUPPLEMENTARY MATERIALS AND TABLES


