# Supplementary material for: Spatial habitats from multiparametric MR imaging are associated with signaling pathway activities and survival in glioblastoma
Source: Oncotarget. 2017 Dec 5;8(68):112992–3001. doi: 10.18632/oncotarget.22947 (PMC5762568; doi:10.18632/oncotarget.22947)
Supplement: Supplementary file 1 [file oncotarget-08-112992-s001.pdf]

Spatial habitats from multiparametric MR imaging are associated with signaling pathway activities and survival in glioblastoma

SUPPLEMENTARY MATERIALS

Supplementary Table 1: Assessing relationship between Habitat proportions and patient OS using multivariate Cox proportional hazards regression analysis, adjusting for covariates age, KPS, tumor volume, and IDH1 mutation

Habitat number	*P* value	Enhancement combination based on the pixel intensity			
		FLAIR	T1	T1C	T2
0	0.447	0	0	0	0
1	0.016	0	0	0	1
2	0.008	0	0	1	0
3	0.385	0	0	1	1
4	0.560	0	1	0	0
5	0.961	0	1	0	1
6	0.137	0	1	1	0
7	0.033	0	1	1	1
8	0.802	1	0	0	0
9	0.521	1	0	0	1
10	0.035	1	0	1	0
11	0.996	1	0	1	1
12	0.689	1	1	0	0
13	0.318	1	1	0	1
14	0.264	1	1	1	0
15	0.965	1	1	1	1

Habitats 1, 2, 7, 10 are significantly associated with OS. Note that only habitats 2, 7, 10 have positive variable importance as well (from random forest analysis). Hence, only those are considered for further interpretation.
